# Apigenin inhibits NF-κB and Snail signaling, EMT and metastasis in human hepatocellular carcinoma

**DOI:** 10.18632/oncotarget.9404

**Published:** 2016-05-17

**Authors:** Yuan Qin, Dong Zhao, Hong-gang Zhou, Xing-hui Wang, Wei-long Zhong, Shuang Chen, Wen-guang Gu, Wei Wang, Chun-hong Zhang, Yan-rong Liu, Hui-juan Liu, Qiang Zhang, Yuan-qiang Guo, Tao Sun, Cheng Yang

**Affiliations:** ^1^ State Key Laboratory of Medicinal Chemical Biology and College of Pharmacy, Nankai University, Tianjin, China; ^2^ Tianjin Key Laboratory of Molecular Drug Research, Nankai University and Tianjin International Joint Academy of Biomedicine, Tianjin, China; ^3^ Department of Pathology, The People's Hospital of Shouguang City, Shouguang, Shandong Province, China

**Keywords:** apigenin, antitumor, EMT, metastasis, HCC

## Abstract

Apigenin is a naturally occurring compound with anti-inflammatory, antioxidant, and anticancer properties. In this study, we investigated the effects of apigenin on migration and metastasis in experimental human hepatocellular carcinoma (HCC) cell lines *in vitro* and *in vivo*. Apigenin dose-dependently inhibited proliferation, migration, and invasion by PLC and Bel-7402 human HCC cells. It also suppressed tumor growth in PLC cell xenografts without altering body weight, thereby prolonging survival. Apigenin reduced Snai1 and NF-κB expression, reversed increases in epithelial-mesenchymal transition (EMT) marker levels, increased cellular adhesion, regulated actin polymerization and cell migration, and inhibited invasion and migration by HCC cells. Apigenin may therefore inhibit EMT by inhibiting the NF-κB/Snail pathway in human HCC.

## INTRODUCTION

Apigenin (4′, 5, 7-trihydroxyflavone, 5, 7-dihydroxy-2-(4-hydroxyphenyl)-4H-1-benzopyran-4-one) is a naturally occurring plant flavone present in many fruits, vegetables and herbs, which has anti-inflammatory, antioxidant, and anticancer effects [1]. In natural sources, apigenin exists as apigenin-7-O-glucoside and as various acylated derivatives [2]. For hundreds of years, apigenin has been utilized as a traditional medicine. For instance, passion flower, which contains high levels of apigenin, has been used effectively to treat asthma, intransigent insomnia, Parkinson's disease, neuralgia, and shingles [3]. In recent years, apigenin has received increasing attention for its antitumor activity.

Several studies have shown that apigenin inhibits tumor cell invasion and metastasis [4–7]. For instance, apigenin inhibits hepatoma cell growth by altering gene expression patterns [8] and inducing cancer cell apoptosis. Liver cancer is a leading cause of cancer mortality worldwide [9]. Although many standard therapies and targeted agents have been developed, tumor recurrence and metastasis remain common in liver cancer. No single medicine can effectively treat human hepatocellular carcinoma (HCC), and intrahepatic and lung metastases readily develop due to epithelial-mesenchymal transition (EMT), a key process supporting tumor metastasis. We therefore investigated the effects of apigenin on EMT. Our findings demonstrate that apigenin reverses EMT via a NF-κB/Snail pathway in human liver cancer.

## RESULTS

### Apigenin reduces cell viability and inhibits migration and invasion in human liver cancer cell lines

**Figure 1:**
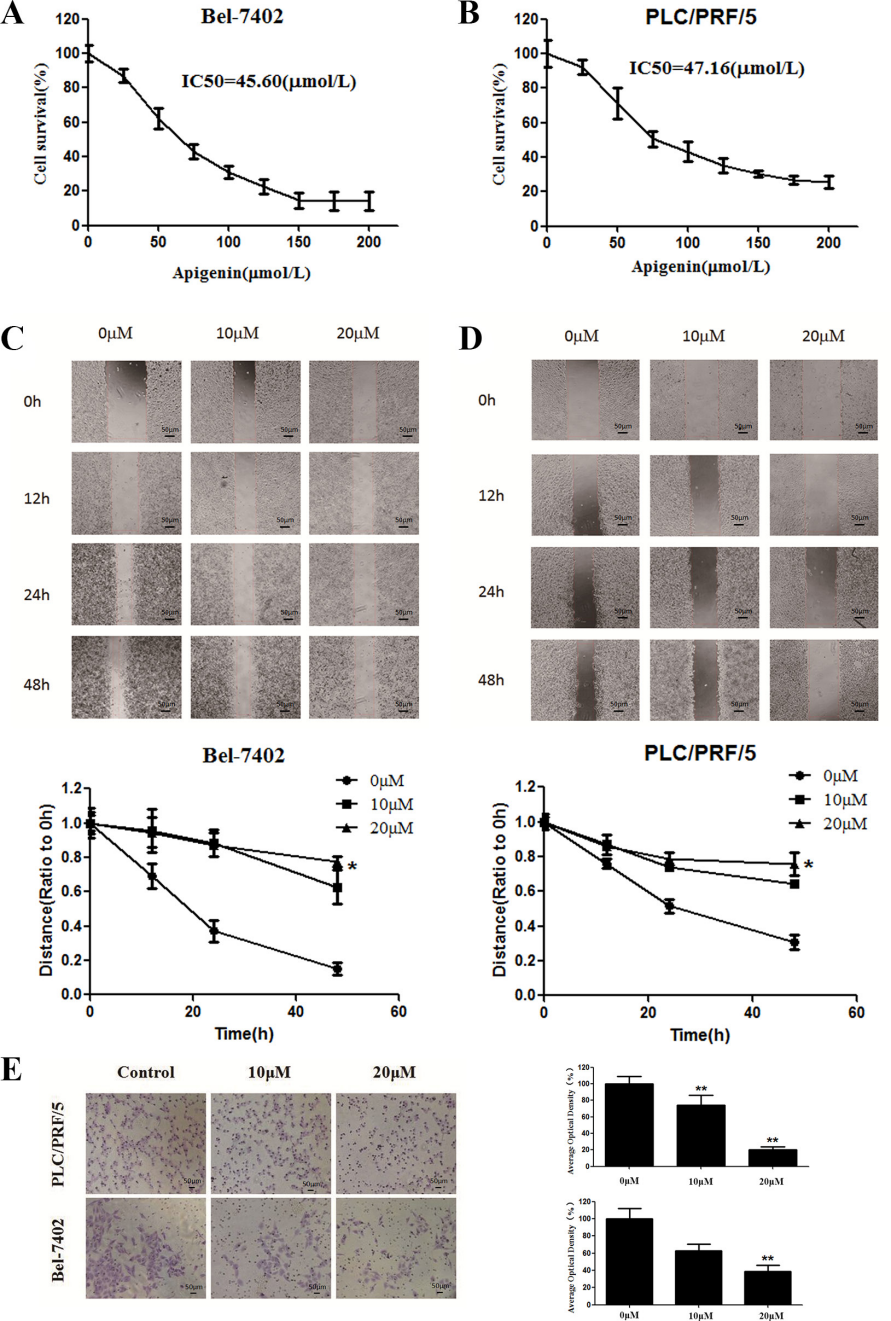
Effects of apigenin on cell viability and migration (**A**) Survival of Bel-7402 cells treated with the indicated amounts of apigenin for 48 h; IC_50_ = 45.60 μM. (**B**) Survival of PLC/PRF/5 cells treated with the indicated amounts of apigenin for 48 h; IC_50_ = 47.16 μM. (**C**) Bel-7402 cell viability was inhibited after re-incubation in medium containing 0, 10, or 20 μM apigenin for 48 h. (**D**) PLC/PRF/5 cell viability was inhibited after re-incubation in medium containing 0, 10, or 20 μM apigenin for 48 h. (**E**) Transwell chambers were utilized for the invasion assay, and images were obtained at 200× magnification. PLC/PRF/5 and Bel-7402 cells were treated with 0, 10, or 20 μM apigenin. Each experiment was performed in triplicate. The results are shown as the means of the three experiments, and the error bars represent the standard deviation (*P < 0.05).

Using an MTT assay, we determined the effect of 48 h treatment with apigenin on cell viability in various cancer cell lines. Apigenin reduced cell viability in a dose-dependent manner, as depicted in Figure 1A and 1B. The half-maximal inhibitory concentrations (IC50) of apigenin after 48 h of treatment in Bel-7402 and PLC cells were approximately 45.60 and 47.16 μM, respectively. The effects of apigenin on cell viability in other hepatocarcinoma cell lines, normal human liver cells, and normal human cells are shown in Figure S1.

Next, we assessed the ability of apigenin to inhibit migration in Bel-7402 and PLC cells using a wound-healing assay. Confluent cells were scraped with a sterile pipette tip, and the remaining cells were allowed to migrate to the resulting gap in the absence or presence of apigenin. Remarkably, after 24 and 48 h treatment, the wound gap was wider in the apigenin-treated groups than in the untreated groups in both cell types (Figure 1C and 1D), indicating that apigenin inhibits the motility of both Bel-7402 and PLC cells. We also conducted this experiment using HMCC-97L, HepG2, SMMC-7721, and LO2 cell lines. Apigenin inhibited migration in these different hepatoma cell lines, but did not affect normal liver cells (LO2 cell lines) (Figure S2).

To investigate whether apigenin inhibits Bel-7402 and PLC cell invasion, we utilized matrigel-coated transwell chambers. Apigenin dose-dependently reduced the number of cell invasions through the matrigel-coated filter in both cell lines as compared to the control group (Figure 1E). Therefore, apigenin markedly inhibited invasion in both Bel-7402 and PLC cells.

### Apigenin alters cellular morphology and reverses changes in EMT biomarkers in human liver cancer cell lines

**Figure 2:**
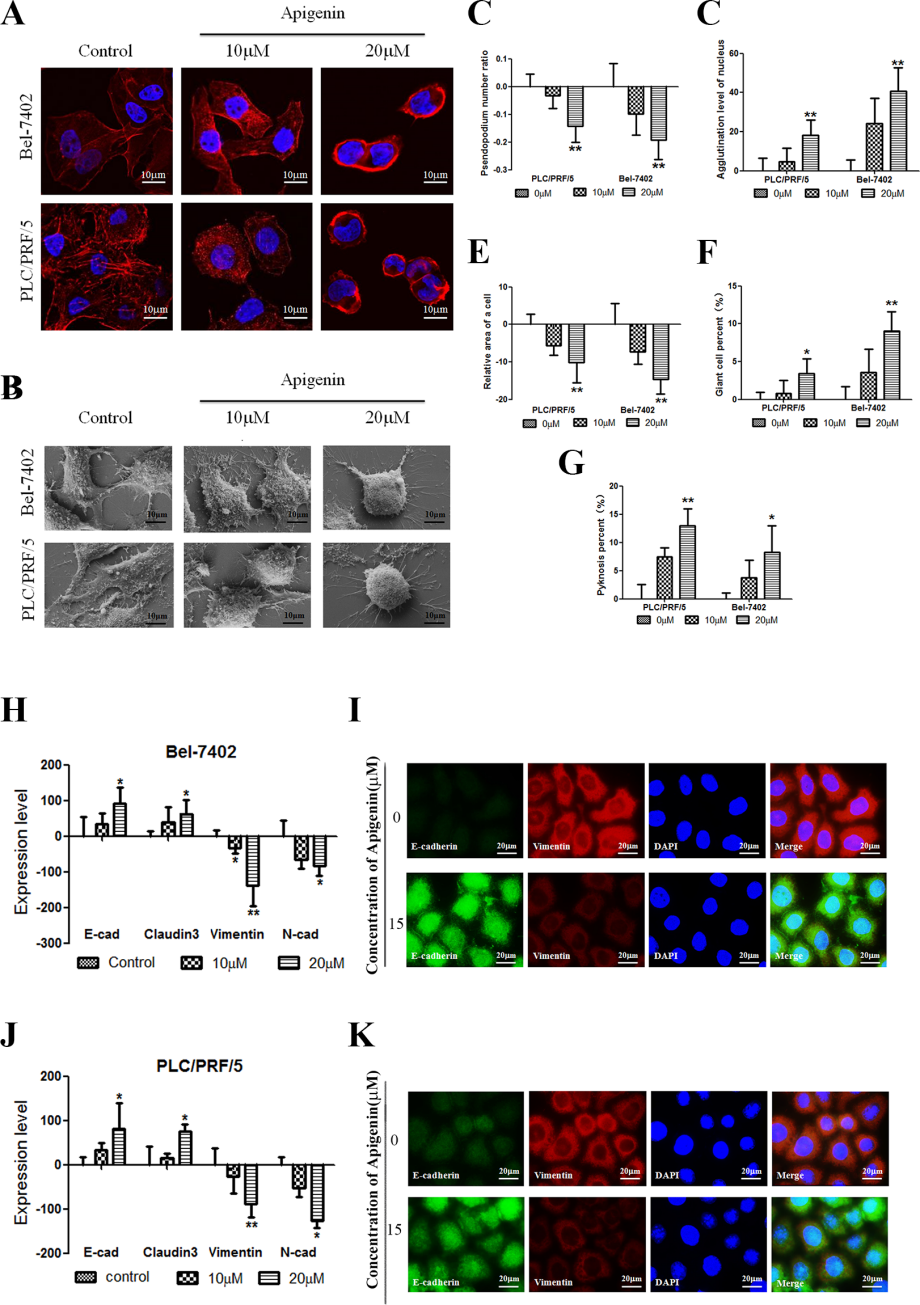
Apigenin alters cellular morphology and reverses changes in EMT biomarkers (**A** and **B**) Confocal images of actin cytoskeleton stained with rhodamine–phalloidin and scanning electron micrographs. Treatment of cancer cells with apigenin led to the agglutination of β-actin, contraction of pseudopodia, cell shrinkage, and changes in cell shape. (**C**–**G**) The number of pseudopodia and cell area decreased when cells were treated with apigenin. The agglutination level and giant and pyknosis cell numbers increased after apigenin treatment. (**H** and **J**) In Bel-7402 and PLC/PRF/5 cells, Vimentin and N-cadherin expression decreased, whereas E-cadherin and claudin-3 expression increased, following apigenin treatment. (**I** and **K**) Typical images of immunofluorescent double staining for E-cadherin and Vimentin in Bel-7402 and PLC/PRF/5 cells. Each experiment was performed in triplicate. The results are shown as the means of the three experiments, and the error bars represent the standard deviation (*P < 0.05).

Morphological features were characterized with a laser scanning confocal microscope, a scanning electron microscope, and HCS. As shown in Figure 2A, cells were stained with a fluorochrome of F-actin. Low doses of apigenin led to the contraction of pseudopodia and changes in microfilament structure. However, high doses of apigenin led to the agglutination of β-actin, cell shrinkage, and changes in cell shape. As shown in Figure 2B, the changes in cellular morphology were similar to those in the cells in Figure 2A. The effect of apigenin on hepatoma and normal liver cellular microtubule morphology is shown in Figure S3. The number of pseudopodia and cell area decreased following apigenin treatment. β-actin agglutination and giant and pyknosis cell numbers increased following apigenin treatment (Figure 2C–2G). We utilized HCS to measure the effects of apigenin on epithelial cell biomarker (E-cadherin and claudin3) and mesenchymal marker (Vimentin and N-cadherin) levels. Bel-7402 and PLC cells were treated with different doses of apigenin for 24 h. Vimentin and N-cadherin levels decreased, whereas E-cadherin and claudin3 levels increased, in response to apigenin treatment in both cell lines (Figure 2H and 2I). Immunofluorescent double staining was performed for E-cadherin and Vimentin and visualized using a fluorescence microscope. Vimentin levels decreased and E-cadherin levels increased in both cell lines, similar to the results from HCS (Figure 2J and 2K). As shown in Figure S5, Western Blotting confirmed these changes in E-cadherin, Claudin3, Vimentin, and N-cadherin levels.

### Apigenin inhibits NF-κB in human liver cancer

**Figure 3:**
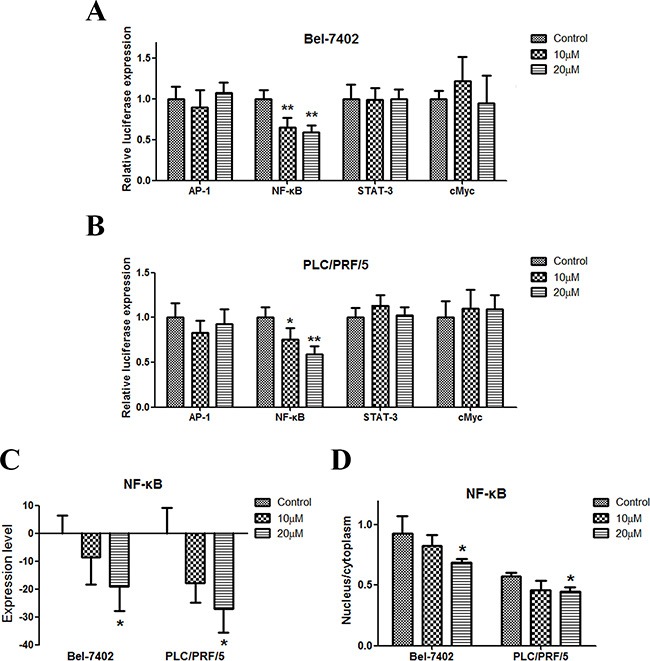
Apigenin inhibits NF-κB in human liver cancer (**A** and **B**) Dual-luciferase assay results for Bel-7402 and PLC/PRF/5 cells suggest that NF-κB is a target gene of apigenin. Apigenin reduced NF-κB expression in the two cell lines. AP-1, STAT3, and cMyc gene expression were not influenced by apigenin. (**C**) Apigenin reduced NF-κB protein levels in the two cell lines. (**D**) Apigenin reduced the nuclear to cytoplasmic protein content ratio in the two cell lines. Each experiment was performed in triplicate. The results are shown as the means of the three experiments, and the error bars represent the standard deviation (*P < 0.05).

We tested the influence of apigenin on the expression of AP-1, NF-κB, STAT-3, and cMyc, which are important signal pathway molecules in cancer cells. The results of the reporter gene assay showed that only NF-κB was downregulated by apigenin (Figure 3A and 3B). NF-κB expression decreased in the two cell lines (Figure 3C). Moreover, the percentage of NF-κB in the nucleus decreased in the two cell lines (Figure 3D and Figure S4).

### Apigenin inhibits snail in human liver cancer

**Figure 4:**
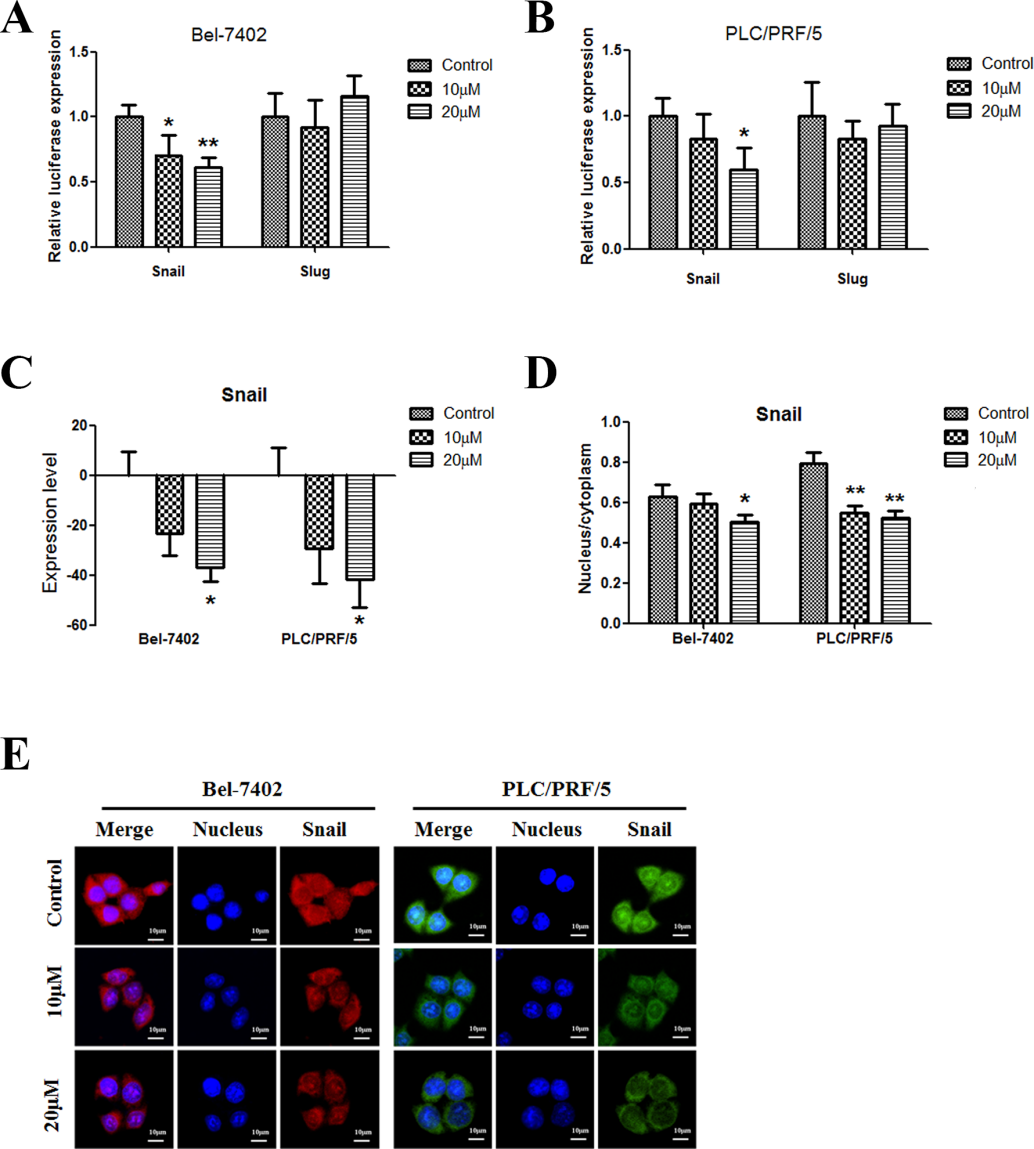
Apigenin inhibits snail in human liver cancer (**A** and **B**) Dual-luciferase assay results for Bel-7402 and PLC/PRF/5 cells suggest that Snail is a target gene of apigenin. Apigenin reduced Snail expression in the two cell lines; Slug expression did not change. (**C**) Apigenin reduced Snail protein levels in the two cell lines. (**D**) Apigenin reduced the nuclear to cytoplasmic Snail protein content ratio in the two cell lines. (**E**) Typical immunofluorescence images of Snail in Bel-7402 and PLC/PRF/5 cells. Each experiment was performed in triplicate. The results are shown as the means of the three experiments, and the error bars represent the standard deviation (*P < 0.05).

Snail and Slug are two members of the Snail family of transcription factors; they play critical roles in EMT. The results of reporter gene assays showed that apigenin inhibited Snail, but not Slug, expression in both cell lines (Figure 4A and 4B); Snail expression decreased in the two cell lines (Figure 4C). Moreover, the percentage of Snail in the nucleus decreased in both cell lines (Figure 4D). Figure 4E shows representative images of Bel-7402 cells and PLC/PRF/5 cells.

### Apigenin exhibits an antitumor effect in a mouse xenograft model

**Figure 5:**
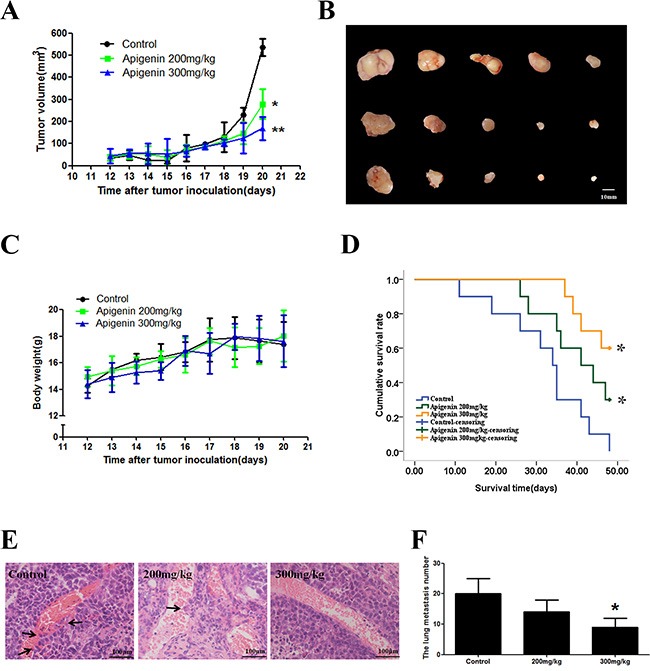
Effect of apigenin on a nude mouse xenograft model Mice were treated with saline or apigenin (200 and 300 mg/kg) for 9 d. (**A**) Changes in the tumor volume of PLC/PRF/5 xenografts. Apigenin reduced tumor volume. (**B**) Apigenin treatment inhibited xenograft growth in a dose-dependent manner. (**C**) Body weights (g) of animals with PLC/PRF/5 xenografts. (**D**) The median survival time of the control xenograft groups was 34.0 ± 2.1 days. The median survival times for low-dose and high-dose apigenin xenograft groups were 39.0 ± 4.0 days and 41 ± 4.7 days, respectively. (**E** and **F**) The numbers of blood metastases in the tumor and tumors that shifted stoves decreased after apigenin treatment. Each experiment was performed in triplicate. The results are shown as the means of the three experiments, and the error bars represent standard deviation (*P < 0.05).

We examined the effects of apigenin on PLC/PRF/5 xenografts in nude mice. Apigenin treatment inhibited tumor growth in a dose-dependent manner with nearly no effect on body weight (Figure 5A to 5C). The median survival time of the apigenin groups increased compared to the control group (Figure 5D). The numbers of blood metastases and of tumors that shifted stoves decreased following apigenin treatment (Figure 5E and 5F).

### Apigenin alters EMT marker levels and inhibits the NF-κB/Snail pathway in cancer tissues

**Figure 6:**
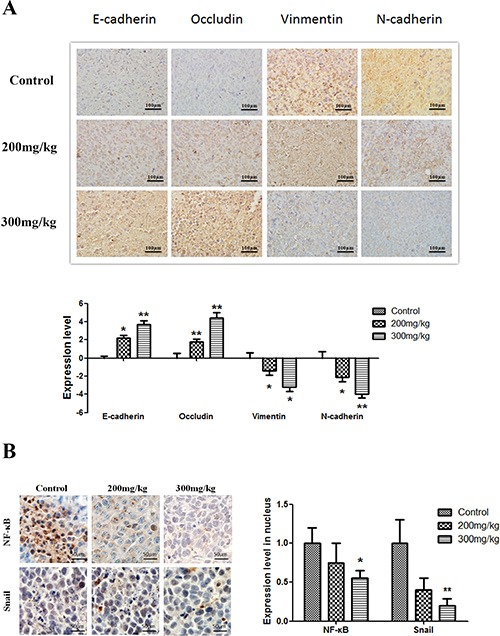
Effect of apigenin on NF-κB, Snail, and EMT protein levels Brown or yellow staining was observed in the cytoplasm or nucleus. (**A** and **B**) Representative photographs of treated and untreated cells. Apigenin treatment increased E-cadherin and Occludin staining and reduced Vimentin and N-cadherin staining compared to sections obtained from control mice. (**C** and **D**) Representative photographs of treated and untreated cells. Nuclear staining decreased in cells treated with apigenin. Each experiment was performed in triplicate. The results are shown as the means of the three experiments, and the error bars represent the standard deviation (*P < 0.05).

Immunohistochemical staining for E-cadherin, Occludin, Vimentin, and N-cadherin showed that apigenin treatment increased E-cadherin and Occludin levels, whereas it decreased Vimentin and N-cadherin levels, in both the membranes and cytoplasm of tumor cells as compared to untreated cells (Figure 6A). In addition, apigenin treatment reduced nuclear immunohistochemical staining for NF-κB and Snail (Figure 6B).

## DISCUSSION

Apigenin has anti-metastatic activity and low cytotoxicity in various cancer cells, but the details of the mechanisms underlying this effect remain unclear [10–12]. Our results show that treatment with apigenin at concentrations ranging from 25–50% of the IC50 value inhibits migration and invasion in HCC cell lines.

In the past decade, the importance of EMT during the progression in various carcinomas, especially HCC, has become increasingly recognized [13]. EMT initiates tumor metastasis and alters cell-cell adhesion [14–16]. The HCC cell lines employed in this study have mesodermal characteristics and readily undergo EMT [17, 18]. Levels of surface EMT markers change during this process, resulting in cellular cytoskeletal rearrangements that favor metastasis [19]. These rearrangements include changes in the distribution of β-actin, number of pseudopodia, and cell morphology. Treat cancer cells with apigenin led to agglutination of β-actin, contraction of pseudopodia, cell shrinkage and changes in cell shape that indicate EMT was inhibited [20].

E-cadherin, claudin3, Vimentin, and N-cadherin (biomarkers of EMT) mediate cell adhesion. Downregulation of E-cadherin and claudin3 expression is necessary to confer metastatic ability to HCC cells [21, 22]. Vimentin is an intermediate filament protein that, along with microtubules and actin filaments, forms the cytoskeleton. Levels of N-cadherin– i.e., neural cadherin, a mesenchymal cadherin associated with EMT – crucially affect cancer progression and metastasis as well as chemotherapy resistance [23]. In the present study, apigenin increased E-cadherin and claudin3 levels and decreased vimentin and N-cadherin levels. These findings suggest apigenin suppresses EMT in HCC cell lines and in mouse xenografts.

Snail, an E-cadherin suppressor, acts primarily as an EMT inducer [24, 25]. Loss of E-cadherin is a hallmark of EMT, and Snail is the most important transcriptional repressor of E-cadherin. The zinc finger domain at the Snail C-terminal binds DNA to the E-box in the E-cadherin promoter region [26]. Snail also downregulates the expression of other epithelial molecules, including Claudins, Occludins and Muc1, and induces expression of genes associated with a mesenchymal and invasive phenotype, such as fibronectin and MMP9 [24]. Our results show that Snail levels and its activity decrease following apigenin treatment in HCC cells. Apigenin also inhibited the relocation of Snail to the nucleus. However, levels of Slug, another member of the Snail family of transcription factors, were not influenced by apigenin in this study.

NF-κB acts to both promote and maintain an invasive phenotype in cells, and functions as an essential mediator of EMT [27]. In this study, we tested the influence of apigenin on the expression of AP-1, NF-κB, STAT-3, and cMyc. Only NF-κB was downregulated by apigenin. Moreover, the NF-κB pathway regulates Snail expression via transcriptional and post-translational mechanisms. NF-κB can bind to the human Snail promoter and increase Snail transcription [28]. Our results show that NF-κB expression and activity decreased following apigenin treatment in HCC cells. Apigenin also inhibited the relocation of NF-κB to the nucleus, similar to Snail. This is consistent with the earlier finding that NF-κB is an upstream regulator of Snail that indirectly mediates EMT and suggests its effect on NF-κB may be crucial to the ability of apigenin to inhibit tumor progression.

In summary, our results imply that apigenin suppresses cancer invasion by inhibiting EMT by suppressing NF-κB-Snail signaling. Our results provide a new mechanistic basis for the therapeutic application of apigenin in HCC patients. Apigenin may be an effective alternative treatment for persistent carcinoma, as well as a new candidate anti-metastasis drug.

## MATERIALS AND METHODS

### Materials

Apigenin was purchased from Aladdin (Los Angeles, USA). Crystal violet and 3-(4, 5-dimethylthiazol-2-y1)-2, 5-diphenyltetrazolium bromide (MTT) were purchased from Sangon Biotech (Shanghai, China). Matrigel and transwell chambers were purchased from BD Biosciences (San Jose, CA, USA). The antibodies for claudin, N-cadherin, vimentin, and E-cadherin were purchased from Affinity Bioreagents (Colorado, USA). An immunofluorescence staining kit with FITC- and TRITC-labeled goat anti-rabbit IgG and cytoskeleton red fluorescent probe ActinRed were purchased from KeyGEN BioTECH (Nanjing, China). Fluorescein isothiocyanate AffiniPure Goat Anti-Rabbit IgG (H + L) secondary antibodies were purchased from EarthOx (San Francisco, CA, USA). A dual luminescence assay kit was purchased from GeneCopoeia (Guangzhou, China).

### Cell culture

The human cell lines, Bel-7402 and PLC, were obtained from KeyGen Biotech (Nanjing, China). The cells were cultured in a medium supplemented with 10% heat-inactivated (56°C, 30 min) fetal calf serum (Hyclone, USA) and maintained at 37°C with 5% CO_2_ in a humidified atmosphere.

### Cell viability assay

Cell viability was determined through MTT assay. Cells (5 × 10^4^ cells/mL) were seeded on 96-well culture plates. After overnight incubation, the cells were treated with various concentrations of apigenin. After 48 h of incubation, cell viability was measured after the addition of 20 μL MTT at 37°C for 4 h. Afterward, 150 μL dimethyl sulfoxide was added to dissolve the formazan crystals. Optical density was measured at 570 nm with a microplate reader (Multiskan^™^ FC, Thermo Scientific, Waltham, MA, USA).

### Wound-healing assay

Bel-7402 and PLC cells were grown on a 35 mm dish to 100% confluence. Cells were starved in media with 0.5% serum and then scratched with sterile pipette tips to form a 100 μm wound. The cells were then cultured in the presence or absence of apigenin in media (1.5% serum) for 24 h. Images of the cells were obtained at 24 and 48 h using a light microscope (Nikon, Japan).

### Invasion assays

Cell invasion assays were performed in a transwell chamber with a polyethylene terephthalate filter membrane containing 8.0 μm pores on 24-well plates (Corning, USA). For cell invasion assays, the filter membranes were coated with Matrigel. Cells (1 × 10^5^ cells/mL) suspended in 200 μL of serum-free medium were seeded into the upper compartment of the transwell chamber. The lower chamber was filled with medium containing 10% fetal bovine serum (as a chemoattractant for migrating and invading cancer cells), and both compartments were filled with various concentrations of apigenin. After incubation for 24 h, the medium in the upper chamber was removed, and the filters were fixed with precooled methanol (−20°C) for 20 min. The cells remaining on the upper surface of the filter membrane were then completely removed by wiping with a cotton swab, and the cells on the opposite surface of the filter membrane were stained with 0.1% crystal violet for 10 min. The invading cells were then visualized and the absorbance of crystal violet dye was measured for each compartment.

### Cytoskeleton staining

Bel-7402 and PLC cells (5 × 10^4^ cells/mL) were seeded on 24-well culture plates on glass slides. After overnight incubation, the cells were treated with various apigenin concentrations. After incubation for 24 h, the cells were washed twice in phosphate-buffered saline (PBS), fixed with 10% formalin in PBS, permeabilized, and blocked with PBS containing NP-40 (0.1%) and bovine serum albumin (BSA) (3%). The cells were then incubated in the same solution containing cytoskeleton red fluorescent probe ActinRed (1:40 dilution) for 20 min at room temperature (25°C). The cells were washed twice in PBS before the glass slides with cells were placed on a microslide with DAPI-Fluoromount-G. The cells were visualized with a laser scanning confocal microscope (Nikon, Japan).

### Immunofluorescent staining

Bel-7402 and PLC cells (4 × 10^4^ cells/mL) were seeded on 96-well culture plates. After overnight incubation, the cells were treated with various concentrations of apigenin. After incubation for 24 h, the cells were washed twice in PBS, fixed with 10% formalin in PBS, permeabilized, and blocked with PBS containing NP-40 (0.1%) and BSA (3%). The cells were then incubated in the same solution containing primary antibodies specific for E-cadherin (1:50 dilution), claudin3 (1:50 dilution), vimentin (1:50 dilution), or N-cadherin (1:50 dilution) for 1 h at room temperature (25°C). The cells were washed four times in PBS and incubated in secondary antibody (1:200 dilution) for 30 min at room temperature (25°C). The cells were then washed four times in PBS and covered with Hoechst 33342 dye for 30 min at room temperature. The cells were again washed four additional times in PBS; thereafter, proteins were visualized with high-content screening (HCS) systems (Thermo Fisher, USA).

### Dual-luciferase assay

Bel-7402 and PLC cells were transfected with dual-reporter constructs using transfection reagents. After changing to fresh medium 24 h after transfection, the cells were treated with various concentrations of apigenin. After 48 h, the culture medium (Snail and Slug) or cell lysate (AP-1, NF-κB, STAT-3 and cMyc) was collected on a 96-well white plate, and luminescence was measured with a luminometer. The details of various transcription factors are shown in Table S1 and Table S2.

### Animal studies

Four- to five-week-old female BALB/c nu/nu mice were maintained in a pathogen-free animal care facility according to institutional guidelines. All animals in this experiment were well taken care of. PLC^EMT^ xenografts of tumors were established by subcutaneous injection of 1 × 10^6^ cells (suspended in PBS) into the flank. One day after tumor cell inoculation, the mice were randomly divided into three groups (*n* = 5). After the tumors reached an approximate volume of 100 mm^3^ (approximately two weeks after injection), the mice in the treatment groups were treated with 200 or 300 mg/kg apigenin via intraperitoneal injection, whereas the mice in the control groups were injected with saline. Body weights were measured at different time points after tumor cell inoculation. Tumor diameters were measured daily, and tumor volumes were calculated according to the formula *V* = ab^2^/2 (a = length of tumor, b = width of tumor). All mice were euthanized seven weeks after treatment, and both xenografts and livers were resected and measured. The xenografts were harvested for histologic examination, and blood-borne metastasis was measured after HE staining.

Another 15 mice were allocated randomly to the three groups as described above (5 mice per group) to measure the survival rates. Each mouse was injected with 1 × 10^6^ cells (suspended in PBS) in the caudal vein. The survival time of each mouse was recorded.

### Immunohistochemical analysis

Fresh tissues from mice were fixed in 4% paraformaldehyde, embedded in paraffin, cut into 4 μm-thick slices, and placed on slides. The tissues were deparaffinized with xylene, dehydrated in decreasing concentrations of ethanol, and subsequently incubated with 3% hydrogen peroxide for 15 min to block endogenous peroxidase activity. For antigen retrieval, tissues were treated with citrate buffered saline (pH 6.0) for 15 min at 95°C. The tissues were incubated with normal goat serum for 20 min at room temperature to block unspecific labeling and then incubated with the following primary antibodies in a humidified chamber overnight at 4°C: rabbit polyclonal anti-E-cadherin (Zhongshan, ready to use), goat polyclonal anti-vimentin (Affinity, dilution 1:50), and rabbit polyclonal anti-MMP-9 (Zhongshan, ready to use). Diaminobenzidine and hematoxylin were utilized for color development and as counterstain, respectively. E-cadherin and vimentin staining were independently evaluated by two investigators. Tumor cells with brown staining of the cytoplasm, nucleus, or membrane were scored as follows: none (0), weak brown (1+), moderate brown (2+), and strong brown (3+). Samples were divided into five classes based on the percentage of tumor cells stained: 0 for no cells, 1 for 1%–25%, 2 for 25%–50%, 3 for 50%–75%, and 4 for > 75%.

### Statistical analyses

All data are expressed as means ± standard deviation. Comparisons between groups were performed by one-way analysis of variance followed by Bonferroni post hoc test (SPSS software package version 17.0, SPSS Inc., Chicago, IL, USA). The level of significance was set at *P* < 0.05.

## SUPPLEMENTARY FIGURES AND TABLES


