# Supplementary material for: Apigenin inhibits NF-κB and Snail signaling, EMT and metastasis in human hepatocellular carcinoma
Source: Oncotarget. 2016 May 17;7(27):41421–31. doi: 10.18632/oncotarget.9404 (PMC5173069; doi:10.18632/oncotarget.9404)
Supplement: Supplementary file 1 [file oncotarget-07-41421-s001.pdf]

# Apigenin inhibits NF-κB and snail signaling, EMT and metastasis in human hepatocellular carcinoma

## Supplementary Materials

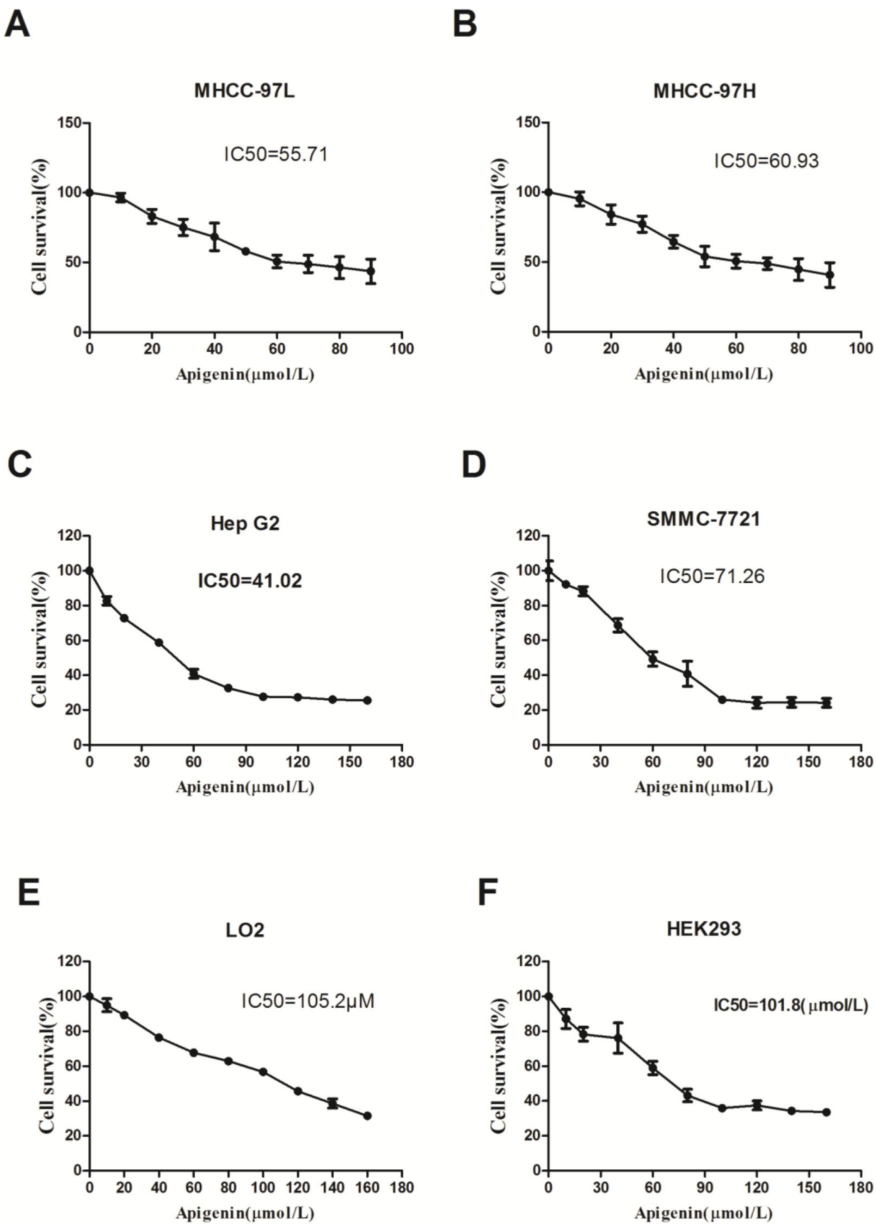


**Supplementary Figure S1: Effects of apigenin on cell viability in hepatocarcinoma cells, normal human liver cells, and normal human cells.** (**A–D**) Survival of different hepatocarcinoma cells treated with the indicated amounts of apigenin for 48 h. (**E**) Survival of LO2 normal human liver cells treated with the indicated amounts of apigenin for 48 h. (**F**) Survival of human embryonic kidney 293 cells treated with the indicated amounts of apigenin for 48 h.

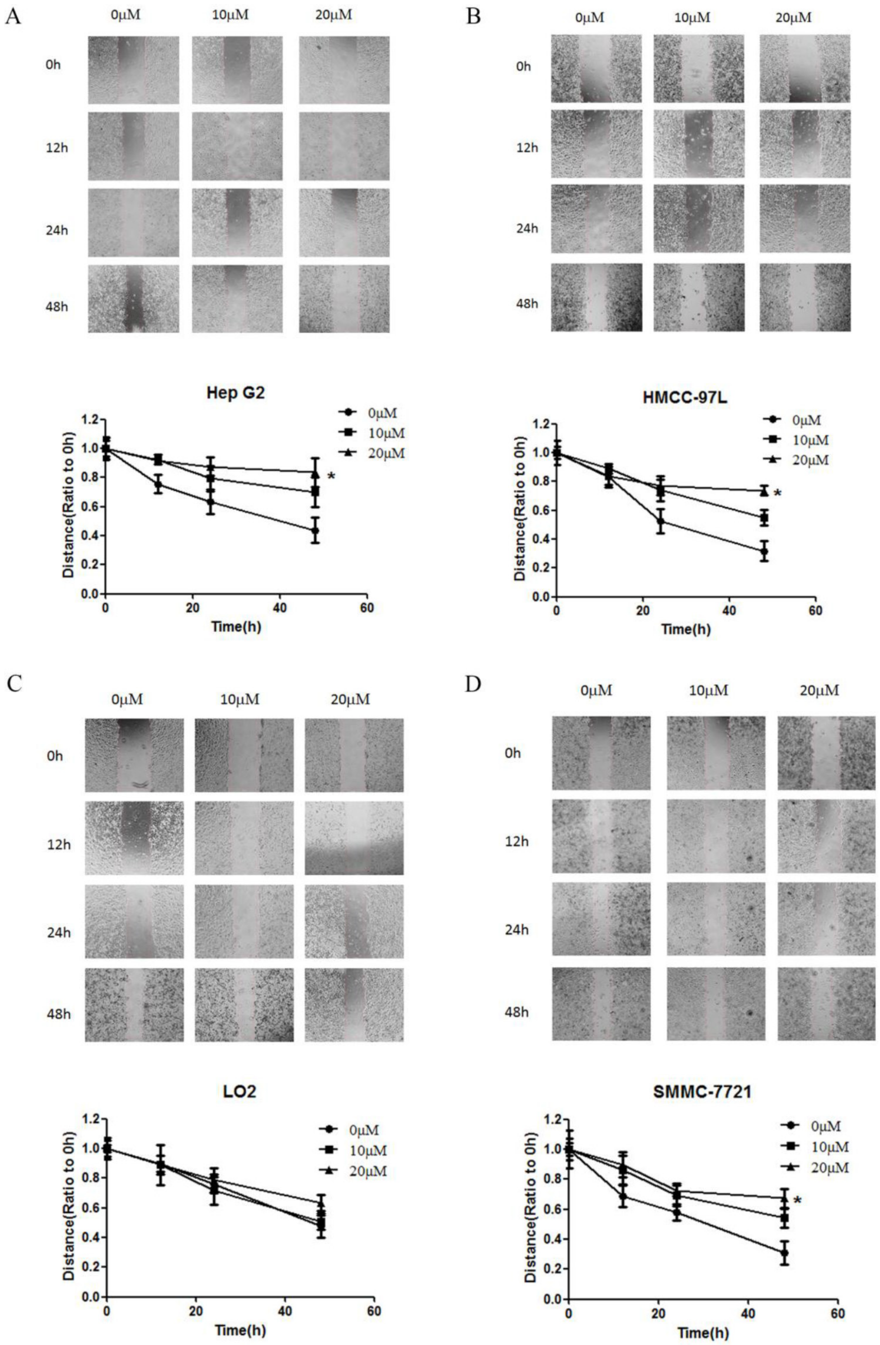


**Supplementary Figure S2: Effects of apigenin on cell migration.** (**A**–**D**) Cells were re-incubated in medium containing 0, 10, or 20 μM apigenin for 48 h; apigenin inhibited migration.

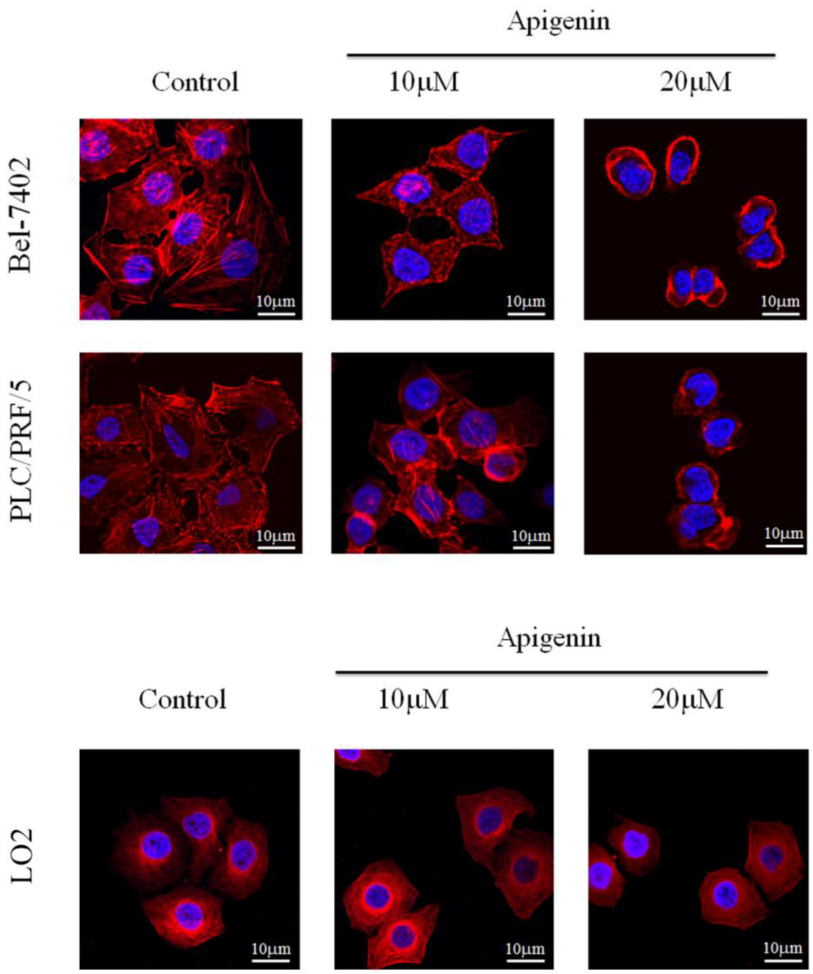


**Supplementary Figure S3: Effect of apigenin on cellular microtubule morphology.**

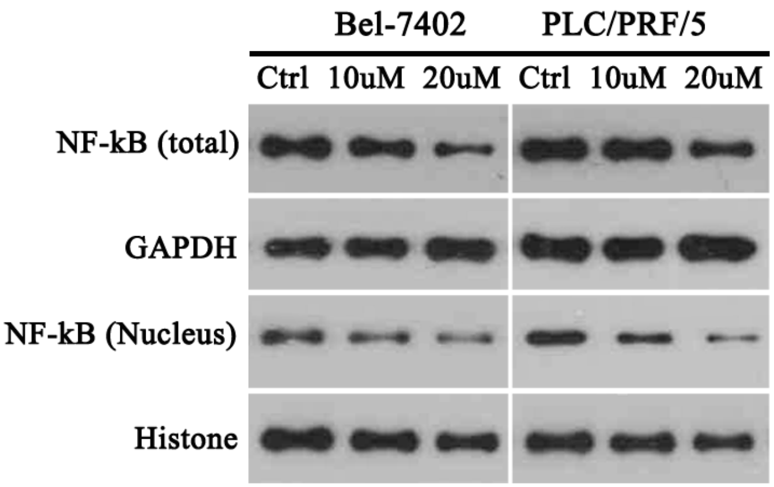


Supplementary Figure S4: Apigenin inhibits NF-κB in human liver cancer.

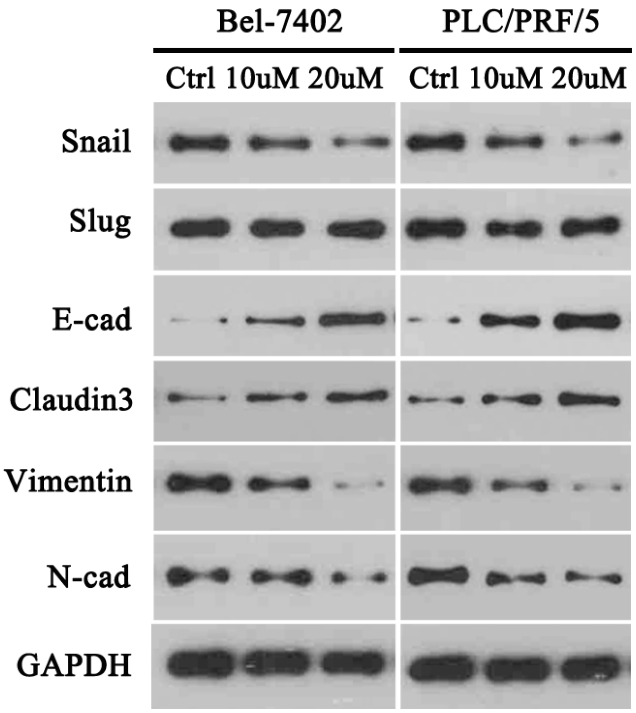


Supplementary Figure S5: Apigenin decreased Snail, Vimentin and N-cadherin levels and increased E-cadherin and Claudin3 levels; Slug levels did not change.

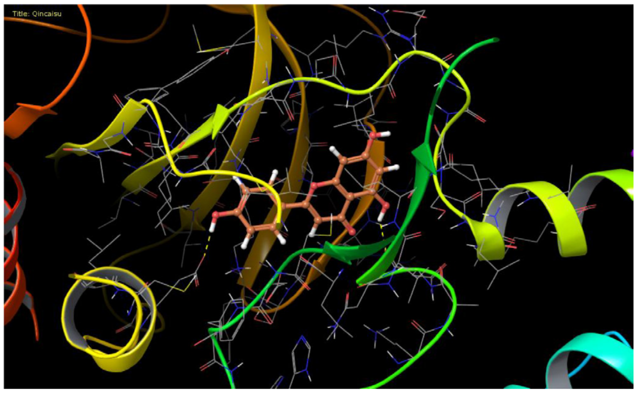


**Supplementary Figure S6: Molecular docking analysis showed that apigenin may target NF-κB-inducing kinase (NIK).**

## Supplementary Table S1: Snail and slug promoter reporter clones

| Promoter reporter clones | Promoter sequence | Vector information |
|---|---|---|
| Snail* | Promoter Length: 1255 bp<br>Sequence length upstream of TSS: 1185 bp<br>Sequence length downstream of TSS: 69 bp | pEZX-PG04 |
| Slug* | Promoter Length: 1399 bp<br>Sequence length upstream of TSS: 1235 bp<br>Sequence length downstream of TSS: 163 bp | |

*The promoter reporter clones were purchased from GeneCopoeia (Guangzhou, China).

## Supplementary Table S2: AP-1, STAT3, NF-κB, cMyc luciferase reporter gene vector

| Reporter gene vector | Response element | Vector information |
|---|---|---|
| pAP1-TA-luc* | AP1 response element 26–67<br>TGACTAATGACTAATGACTAAT<br>GACTAATGACTAATGACTAA | pGL6-TA |
| pSTAT3-TA-luc* | STAT3 response element 32–86<br>TGCTTCCCG AACGTTGCTT<br>CCCGAACGTT GCTTCCCGAA<br>CGTTGCTTCC GAACG | pGL6-TA |
| pNFκB-TA-luc* | NFκB response element 26–65<br>GGGAATTTCCGGGAATTTCCGGGAATTT<br>CCGGGAATTTCC | pGL6-TA |
| pcMyc-TA-luc* | cMyc response element 26–61<br>CACGT GCACGTGCAC GTGCACGTGC<br>ACGTGCACGT | pGL6-TA |
| pRL-TK# | HSV-TK promoter 7–759 | pRL |

*The reporter gene vector were purchased from Beyotime Biotechnology (Shanghai, China).
#The reporter gene vector was purchased from Promega.
